# Association of vitamin D in individuals with periodontitis: an updated systematic review and meta-analysis

**DOI:** 10.1186/s12903-023-03120-w

**Published:** 2023-06-13

**Authors:** Fangfang Liang, Yuanzhu Zhou, Zhenyu Zhang, Zheng Zhang, Jing Shen

**Affiliations:** 1 School of Medicine, Tianjin Stomatological Hospital, Nankai University, Tianjin, 300000 China; 2 Tianjin Key Laboratory of Oral and Maxillofacial Function Reconstruction, Tianjin, 300041 China; 3 The School of Pharmacy, Jiamusi University, Jiamusi, 154007 China

**Keywords:** Vitamin D, 25(OH)D, Periodontitis, Scaling and root planning, Meta-analysis

## Abstract

**Background:**

There are differences in vitamin D levels between periodontitis and healthy individuals, but the effect of vitamin D on periodontitis is controversial. The purpose of this Meta-analysis is twofold: (1) compare vitamin D levels in individuals with or without periodontitis; (2) assess the effects of vitamin D supplementation during scaling and root planing (SRP) on periodontal clinical parameters in individuals with periodontitis.

**Methods:**

A systematic search was conducted in five databases (PubMed, Web of Science, MEDLINE, EMBASE, and Cochrane library), published from the database inception to 12 September 2022. The Cochrane Collaboration Risk of bias (ROB) assessment tool, the risk of bias in non-randomized studies of intervention (ROBINS-I) tool, the Newcastle–Ottawa Quality Assessment Scale (NOS), and Agency for Healthcare Quality and Research (AHRQ) were used to evaluate randomized controlled trial (RCT), non-RCT, case–control study, and cross-sectional study, respectively. Statistical analysis was performed using RevMan 5.3 and Stata 14.0 software, with weighted mean difference (WMD), standardized mean difference (SMD) and 95% confidence intervals (CI) as the effect measures, and heterogeneity was tested by subgroup analysis, sensitivity analysis, Meta-regression.

**Results:**

A total of 16 articles were included. The results of Meta-analysis showed that periodontitis was associated with lower serum vitamin D levels compared to normal population (*SMD* = -0.88, 95%*CI* -1.75 ~ -0.01, *P* = 0.048), while there was no significant difference in serum or saliva 25(OH)D levels between periodontitis and normal population. Additionally, the Meta-analysis showed that SRP + vitamin D and SRP alone had a statistically significant effect on serum vitamin D levels in individuals with periodontitis (*SMD* = 23.67, 95%*CI* 8.05 ~ 32.29, *P* = 0.003; *SMD* = 1.57, 95%*CI* 1.08 ~ 2.06, *P* < 0.01). And SRP + vitamin D could significantly reduce clinical attachment level compared to SRP alone (*WMD* = -0.13, 95%*CI* -0.19 ~ -0.06, *P* < 0.01), but had no meaningful effect on probing depth, gingival index, bleeding index, respectively.

**Conclusion:**

The evidence from this Meta-analysis suggests that the serum vitamin D concentration of individuals with periodontitis is lower than that of normal people, and SRP along with vitamin D supplementation has been shown to play a significant role in improving periodontal clinical parameters. Therefore, vitamin D supplementation as an adjuvant to nonsurgical periodontal therapy has a positive impact on the prevention and treatment of periodontal disease in clinical practice.

**Supplementary Information:**

The online version contains supplementary material available at 10.1186/s12903-023-03120-w.

## Introduction

Periodontitis is a chronic infectious disease caused by microorganisms in dental plaque, which can lead to inflammation and the progressive destruction of the periodontal support tissues [1, 2]. A guide issued by the World Health Organization (WHO) in 2021 showed that more than 3.5 billion people suffered from oral disease in 2017, and severe periodontal disease, which is estimated to be detrimental to 796 million people, especially the elderly, has emerged as the leading cause of tooth loss [3, 4]. The reason is that bacteria in periodontal pockets can induce an immune response that leads to gingival inflammation, periodontal pocket formation, attachment loss, and alveolar bone resorption, ultimately resulting in tooth loss and oral dysfunction [5, 6]. In addition, evidence has shown that the potential risk factors for periodontitis include certain systemic factors, such as hormones, diabetes, stress, genetic susceptibility, tobacco use, alcohol consumption, dietary patterns, and reduced intake of certain nutrients apart from oral factors [7, 8].

Vitamin D is an essential nutrient and precursor hormone that plays an important role in numerous biochemical functions in the body [9]. It is a lipid-soluble vitamin that is mainly ingested in the form of vitamin D_2_ and D_3_ via dietary intake and exposure to sunlight. Both forms of vitamin D are metabolized and activated to 25-hydroxyvitamin D (25(OH)D) in the liver, and subsequently converted to the active form of vitamin D, 1, 25-dihydroxyvitamin D_3_ (1,25(OH)2D3), in the kidney. As the main circulating metabolite in the blood, 25(OH) D is the most representative indicator of vitamin D storage in the human body [10, 11]. Vitamin D deficiency has become a significant public health issue worldwide [12]. Studies have found that low levels of vitamin D can lead to an increased risk of periodontitis [13]. This may be due to vitamin D playing an important role in maintaining calcium and bone homeostasis as well as immune function, it is inferred that vitamin D may affect the development of periodontitis by the regulation of bone metabolism and immunological reaction [14].

Previous studies have compared vitamin D levels in individuals with and without periodontitis, however, the results remain controversial [15–17]. Most studies have shown that periodontitis was associated with lower vitamin D levels compared with non-periodontitis [18–20], while other studies have revealed no correlation among them [21, 22]. In addition, the existing systematic reviews of vitamin D and periodontitis mainly focus on polymorphisms of the vitamin D receptor with periodontal susceptibility [23, 24]. Lack of studies on the quantitative synthesis of vitamin D in individuals with and without periodontitis, and regarding the recent increase in studies of vitamin D as an adjunct to scaling and root planing (SRP) on periodontal outcome indicators, both observational and interventional. Hence, the aim of this Meta-analysis is twofold, to compare vitamin D levels in individuals with or without periodontitis, and to evaluate the effects of SRP + vitamin D on periodontal clinical parameters in individuals with periodontitis.

## Materials and methods

The systematic review and Meta-analysis was reported by following the Preferred Reporting Items for Systematic Reviews and Meta-Analysis (PRISMA) guideline.

### Search strategy and data sources

Five electronic databases including PubMed, Web of Science, MEDLINE, EMBASE, and the Cochrane library were searched from the inception of the database until september 12, 2022. And the search was carried out by means of subject headings combined with free text words to encompass all articles related to administration of vitamin D and periodontal non-surgical treatment. The specific search strategy was (“periodontitis OR periodontal disease* OR periodontal OR gum disease* OR gingivitis OR chronic periodontitis OR periodontal infection* OR periodontal health”) and (“vitamin D OR 25-hydroxy-vitamin OR Twenty five-hydroxy vitamin D OR 25OHD OR 25-hydroxyvitamin D OR 25(OH)D OR calcitriol OR vitamin D supplementation OR vitamin D deficiency OR vitamin D receptor OR VDR”). In addition, the reference lists of all selected articles were also reviewed for potentially relevant studies.

### Inclusion and exclusion criteria

Inclusion criteria: (a) Type of studies: human experimental studies (randomized controlled trial (RCT)/non-RCT/observational studies). (b) Subjects: individuals with periodontitis. (c) Intervention or exposure factors: SRP or SRP + vitamin D. (d) Outcome measures: periodontal clinical parameters, including probing depth (PD), clinical attachment level (CAL), gingival index (GI), and bleeding index (BI); vitamin D indicators, including serum/saliva vitamin D levels or serum/saliva levels of 25(OH)D.

Exclusion criteria: (a) Animal studies or vitro studies. (b) Review or Meta-analysis. (c) Case reports or conference papers. (d) Duplicate publication. (e) Combined with other systemic diseases. (f) Pregnant and lactating women. (g) Qualitative research. (h) Incomplete data and the original articles could not be found.

### Data extraction

Based on the inclusion and exclusion criteria, two reviewers independently screened the literature and extracted the data (FF. Liang & YZ. Zhou). In the event of disagreement, a third author was consulted for confirmation (Z. Zhang). The extracted data included: first author, publication year, study time, country, age, gender, sample size, study design, periodontitis diagnostic criteria, periodontitis severity, interventions, influence/ confounding factor, follow-up time, and outcome.

### Quality and risk-of-bias assessments

Two researchers independently evaluated and cross-checked the literature (FF. Liang & YZ. Zhou), and the Cochrane Collaboration Risk of bias (ROB) assessment tool was used for RCT. It consists of six aspects with seven elements: selection (random sequence production and allocation concealment), implementation (blinding of participants and personnel), measurement (blinding of outcome assessment), follow-up (incomplete outcome data), report (selective outcome reporting) and other bias, every item was classified as "low risk of bias", "high risk of bias" and "unclear" [25]. The risk of bias in non-randomized studies of intervention (ROBINS-I) tool was used for assessing non-RCT. It included seven dimensions, pre-intervention (confounding bias, selection of participants bias), in-intervention (intervention classification bias), and post-intervention (deviation from intended intervention bias, missing data bias, outcome measurement bias, and selection of reported result bias), every item was classified as “low risk”, “moderate risk”,“serious risk”, “critical risk” and “no information” [26].The NOS was used for case–control studies. It is an 8-items scale with 3 dimensions including selection, comparability, and exposure, and the maximum score of the scale is nine [27]. The AHRQ composed of 11 items was used to assess the quality of cross-sectional studies, and responses to each item were "yes," "no," or "unclear," and only one point was scored for yes, and all other responses were 0. A third researcher was consulted in case of disagreement (Z. Zhang).

### Statistical analysis

Revman 5.3 software (Review Manager (RevMan), The Nordic Cochrane Centre, The Cochrane Collaboration, Copenhagen) and Stata 14.0 software (StataCorp, College Station, TX, USA) were used to statistical analysis. Categorical data were combined by relative risk (RR) or odds ratio (OR), and quantitative data were combined by weighted mean difference (WMD) or standardized mean difference (SMD). Heterogeneity was tested by *Q* test and *I*^*2*^ analysis. In the chi-square test, *P* ≤ 0.10 was considered as heterogeneity. In the *I*^2^ test, when the *I*^2^ value was 25%, 50% and 75%, it was suggested low, moderate and high heterogeneity among studies [28]. According to the Cochrane handbook, significant heterogeneity was considered to exist if *I*^2^ ≥ 50% [29]. Therefore, in the present study, if *P* < 0.10 and *I*^2^ < 50%, it was considered that there was no significant heterogeneity, the fixed effects model was used for analysis. On the contrary, the random effect model was used to merge, and further subgroup analysis and sensitivity analysis were used to find the source of heterogeneity.

## Results

### Literature search

**Fig. 1:**
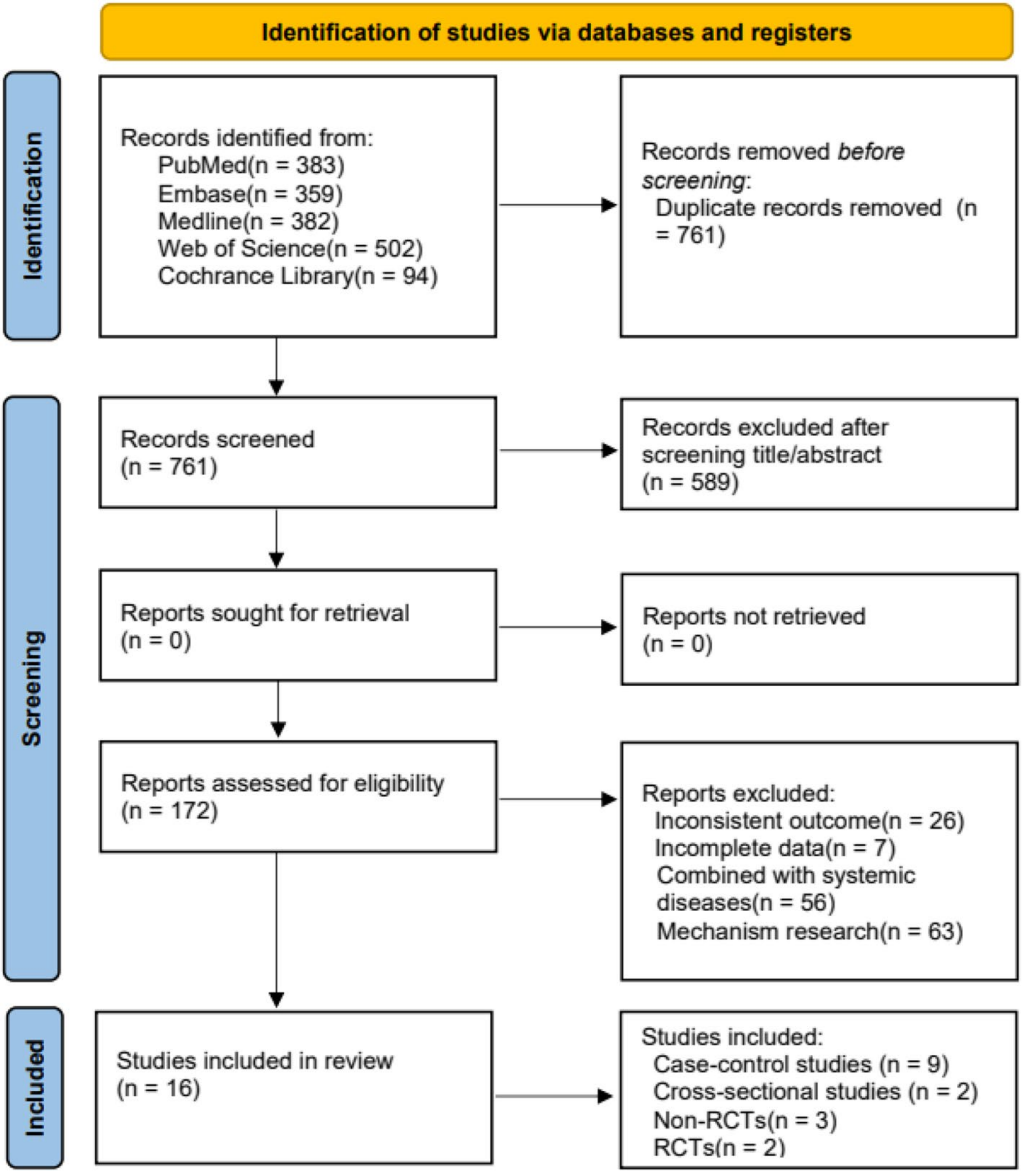
Flow diagram of the study selection

One thousand seven hundred twenty literatures were retrieved from five electronic databases, and 16 literatures [17, 22, 30–43] were finally included. The literature screening process is shown in Fig. 1.

### Study characteristics

A total of 16 articles [17, 22, 30–43] with 1455 subjects were included in this study. There were five intervention studies (two RCTs, three non-RCTs) [33, 36, 39–41], nine case–control studies [17, 22, 30–32, 34, 35, 38, 43], and two cross-sectional studies [37, 42]. The basic characteristics of the included literature are shown in Tables 1 and 2.

**Table 1:** Basic information of the included literature

Study	Study time	Country	Age	Gender (male/female)	No. of case/control	Sample size	Study design
Olszewska-Czyz, I 2022 [17]	NM	Poland	25–62	49/51	50/50	100	Case–control
Costantini, E 2020 [22]	NM	Italy	25–60	14/28	21/21	42	Case–control
Abreu, O 2016 [30]	2014.05–2014.07	Puerto Rican	35–64	10/28	19/19	38	Case–control
Alzahrani, A 2021 [31]	2020.01–2020.11	Saudi Arabia	40.01 ± 7.73	46.3%/53.7%	60/63	123	Case–control
Anbarcioglu, E 2019 [32]	2012–2013	Turkey	21–47	58/71	47/55/27	129	Case–control
Gao, W 2020 [33]	2014.12–2015.06	China	30–70	178/182	120/120/120	360	RCT
Ketharanathan, V 2019 [34]	NM	Tamil and Norway	30–70	92/0	44/48	92	Case–control
Laky, M 2017 [35]	2008–2010	Vienna	28–42	20/38	29/29	58	Case–control
Liu, K 2010 [36]	2006.11–2007.02	China	16–34	8/11	19	19(12)	Non-RCT
Miley, D 2009 [37]	2007.06—2008.02	America	50–80	6/16/17/12	23/28	51	Cross-sectional
Miricescu, D 2014 [38]	NM	Romania	51.26 ± 7.4	16/34	25/25	50	Case–control
Pai, S 2021 [39]	NM	India	30–60	NM	30/30	60	Non-RCT
Perayil, J 2015 [40]	2012.11—2013.12	India	35–55	NM	36/41	77	Non-RCT
Perić, M 2020 [41]	2017.04–2018.10	Brussels	NM	19/7	12/14	26(21)	RCT
Pradhan, S 2021 [42]	2019.02–2019.08	Nepal	25–54	56/24	40/40	80	Cross-sectional
Rafique, S 2019 [43]	NM	Pakistan	18–40	44%/56%	75/75	150	Case–control

*NM* not mentioned, *RCT* randomized controlled trial, *Non-RCT* non-randomized controlled trial

**Table 2:** Clinical characteristics of the included literature

Study	Periodontitis diagnostic criteria	Periodontitis severity	Interventions Control Treatment			Influence/confounding factor	Follow-up time	Outcome
Olszewska-Czyz, I 2022 [17]	2017 Classification of Periodontal Disease	I to IV	NM			Age, Sex, BMI	NM	Vitamin D3 and Clinical Parameters
Costantini, E 2020 [22]	2017 World Workshop on the basis of clinical data	II to IV	NA			NM	NM	PD, CAL, 25(OH)D
Abreu, O 2016 [30]	PD and AL at 6 sites on all teeth	Moderate to severe	NA NM			Socio-economic status and Physical activity	NM	25 (OH)D, PD, AL
Alzahrani, A 2021 [31]	HANES/AAP/CDC	Severe or moderate	NM			Age, Gender, and BMI	NM	25(OH)D, Odds of periodontitis
Anbarcioglu, E 2019 [32]	PPD > 4 mm, AL of > 3 mm	NM	NM			PTH and ALP	NM	PD, CAL, BOP, PI, GI, 1,25(OH)2D
Gao, W 2020 [33]	At least six sites with PD ≥ 6 mm, AL ≥ 4 mm	Moderate to severe	SRP	SRP + 2000 IU/d VD SRP + 1000 IU/d VD		NM	3 months	PD,CAL,BI,25(OH)D
Ketharanathan, V 2019 [34]	CAL ≥ 6 mm in 2 or more teeth and one or more sites PPD ≥ 5 mm	NM	NM			Smoking, Age	NM	Vitamin D
Laky, M 2017 [35]	A minimum of five teeth with a PPD ≥ 5 mm	NM	NM			NM	NM	25(OH)D, Vitamin D,PPD, CAL
Liu, K 2010 [36]	1999 Classification of Periodontal Diseases	NM	Before SRP		After SRP	NM	6 months	25(OH)D3, PD, AL, BI
Miley, D 2009 [37]	At least two interproximal sites with 3 mm CAL	Moderate to severe	NM			Race,Smoking, Gender, and Sun exposure	NM	PD, AL, GI
Miricescu, D 2014 [38]	Six sites PD ≥ 4 mm; bone loss higher than 30%	NM	NA			Smoke, Age	NM	PD,PI,GI,25(OH)D
Pai, S 2021 [39]	Involvement of (> 30% sites) CAL (3–4 mm)	Moderate to severe	Before SRP		After SRP	NM	3 months	GI, PI, CAL, PD, Vitamin D
Perayil, J 2015 [40]	One or more with chronic periodontitis, AL of 3-4 mm	Moderate	SRP		SRP + VD	NM	3 months	Vitamin D, GI, PPD, CAL
Perić, M 2020 [41]	AAP	NM	SRP		SRP + VD	Age,Gender,Smoking	6 months	Vitamin D, PPD
Pradhan, S 2021 [42]	1999 classification of periodontal disease	NM	NM			NM	NM	Vitamin D
Rafique, S 2019 [43]	CAL ≥ 1 mm at two or more nonadjacent sites	Mild to severe	NM			Age and Gender	NM	GI, PI, CAL, PPD,25(OH)D

*AAP* American Academy of Periodontology, *CDC* Centers for Disease Control and Prevention, *HANES* Health and Nutrition Examination Survey, *NM* not mentioned, (P)PD(mm):(pocket)probing depth, *CAL(mm)* clinical attachment level, *AL(mm)* attachment loss, *BI* bleeding index, *BMI* body mass index, *SRP* scaling and root planing, *GI* Gingival index, *PI* Plaque index, BOP:Bleeding on Probing

### Quality assessment

**Fig. 2:**
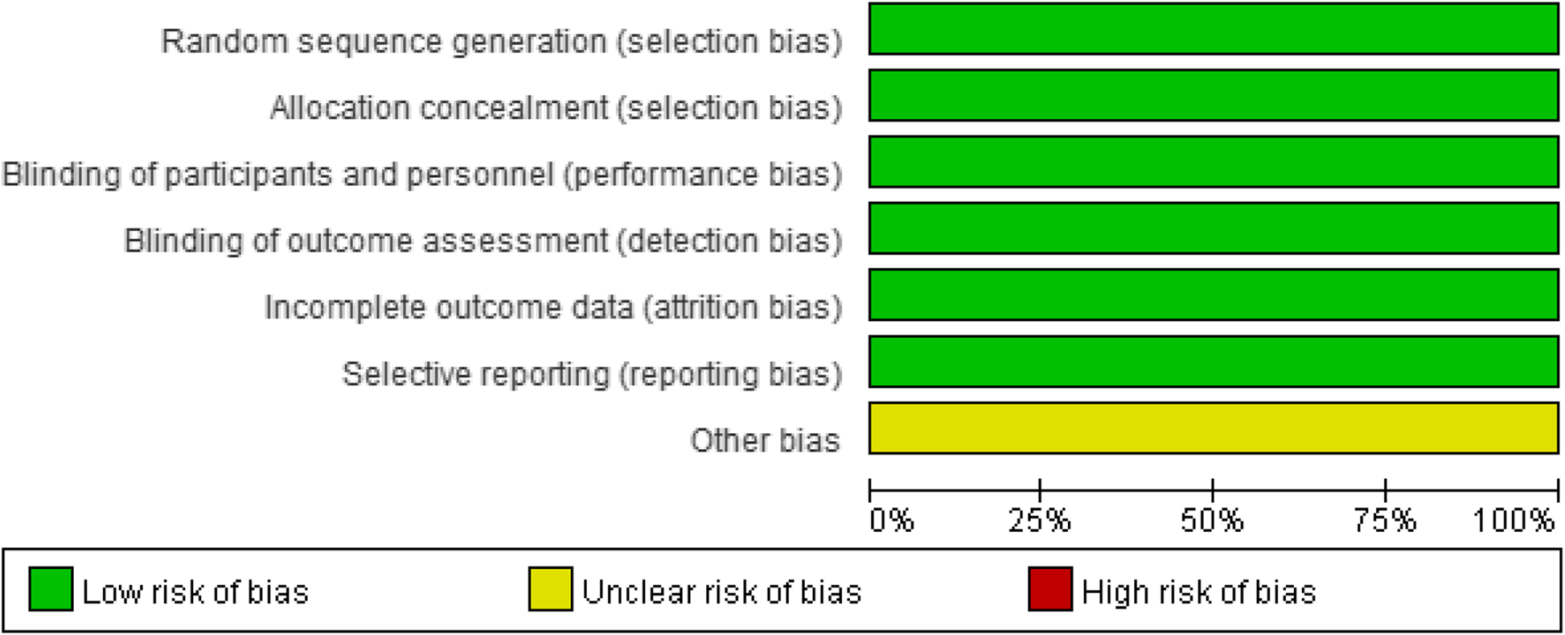
Risk of bias for five intervention studies

**Fig. 3:**
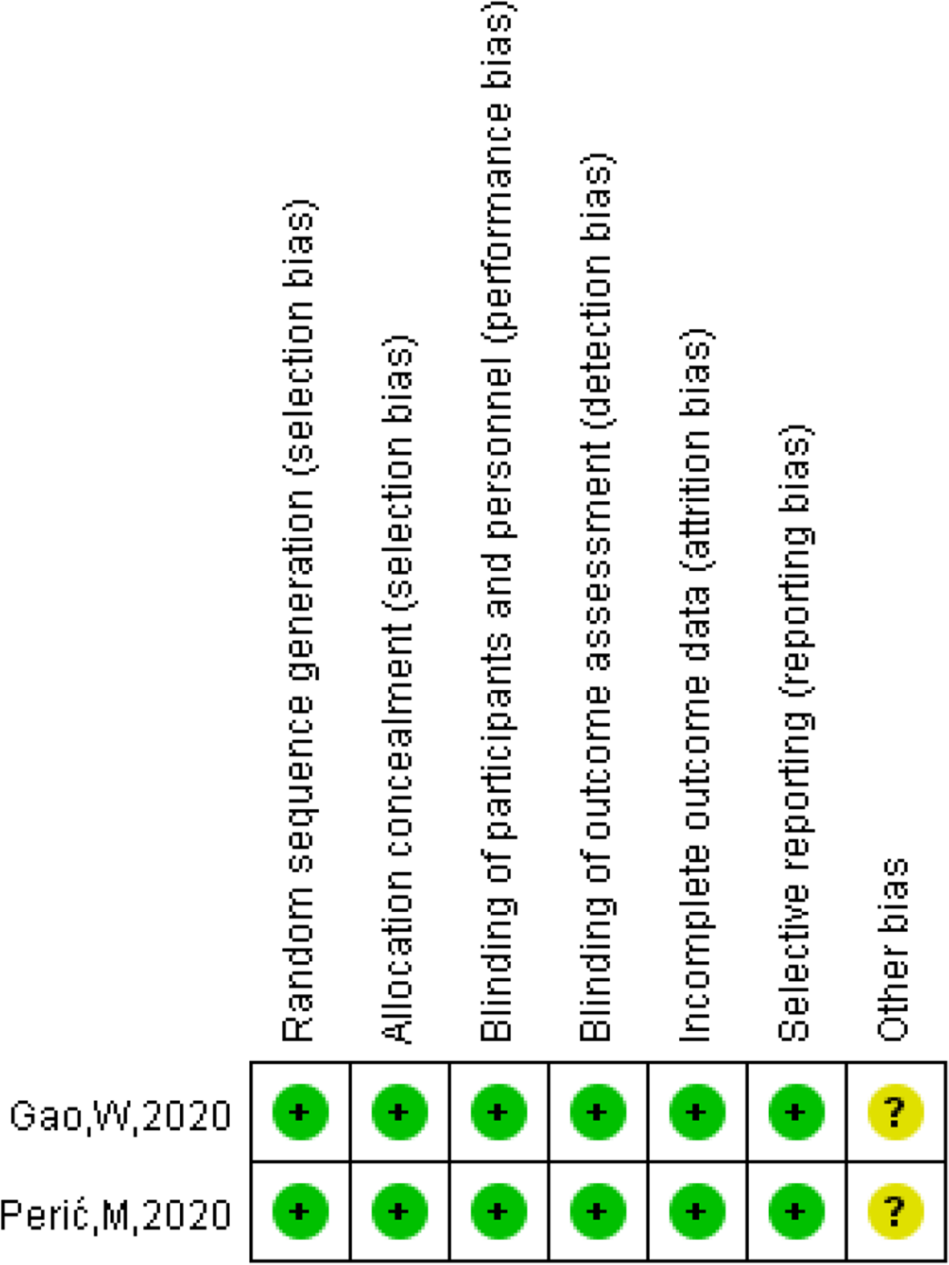
Risk of bias summary for five intervention studies

The NOS scores of the nine case–control studies [17, 22, 30–32, 34, 35, 38, 43] ranged from 7 to 9, and the AHRQ scores ranged from 8 to 9 in the two cross-sectional studies [37, 42]. The two RCTs [33, 41] elaborated on the specific methods of randomization and allocation concealment in detail, and had a low risk of of bias for blinding of participants, personnel, and outcome assessors. Meanwhile, three non-RCTs [36, 39, 41] had a low risk of bias in terms of selection of participants, intervention classification, outcome measurement and selection of reported result (Figs. 2, and 3 and Supplementary Material Table S1, S2, S3).

### Comparison of Vitamin D levels in individuals with and without periodontitis

**Fig. 4:**
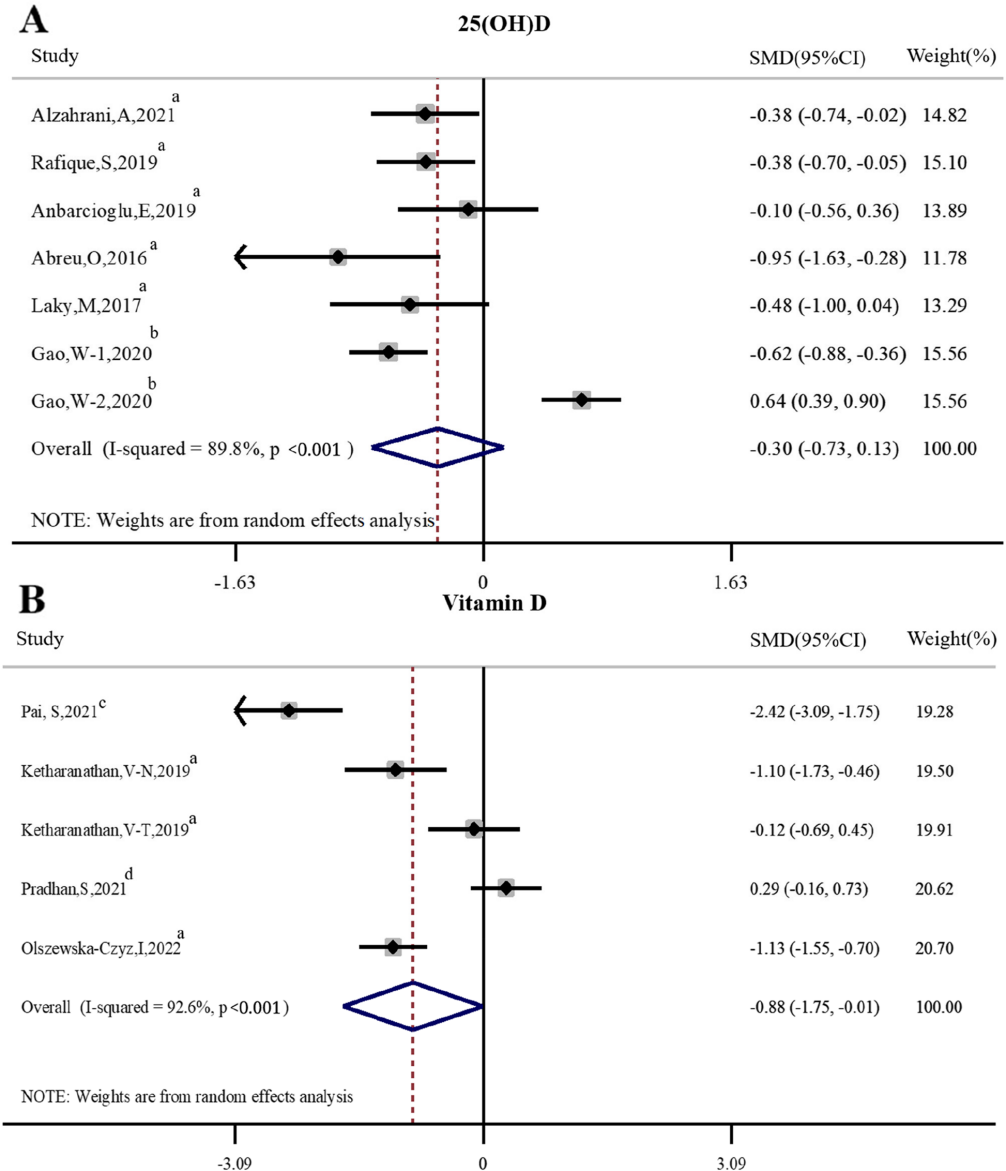
Forest plot of studies evaluating serum 25(OH)D/vitamin D levels in individuals with and without periodontitis. **A** Serum 25(OH)D levels. **B** Serum vitamin D levels. Gao, W-1: Gao, W-2000 IU/d vitamin D; Gao, W-2: Gao, W-1000 IU/d vitamin D; Ketharanathan,V–N: Ketharanathan,V-Norwegian; Ketharanathan,V-T: Keth- aranathan, V-Tamil; SMD: standardized mean difference; a: case–control study; b: RCT; c: Non-RCT; d: cross-sectional study

As shown in Fig. 4. Six articles [30–33, 35, 43] compared the difference in serum 25(OH)D levels between periodontitis and healthy individuals, and serum 25(OH)D levels in periodontitis has no significant difference from the normal population (*SMD* = -0.30, 95%*CI* -0.73 ~ 0.13, *P* = 0.17). At the same time, four articles [17, 34, 39, 42] compared the difference in serum vitamin D levels between periodontitis and healthy individuals, and periodontitis was found to be associated with lower serum vitamin D levels compared to normal population (*SMD* = -0.88, 95%*CI* -1.75 ~ -0.01, *P* = 0.048).

**Fig. 5:**
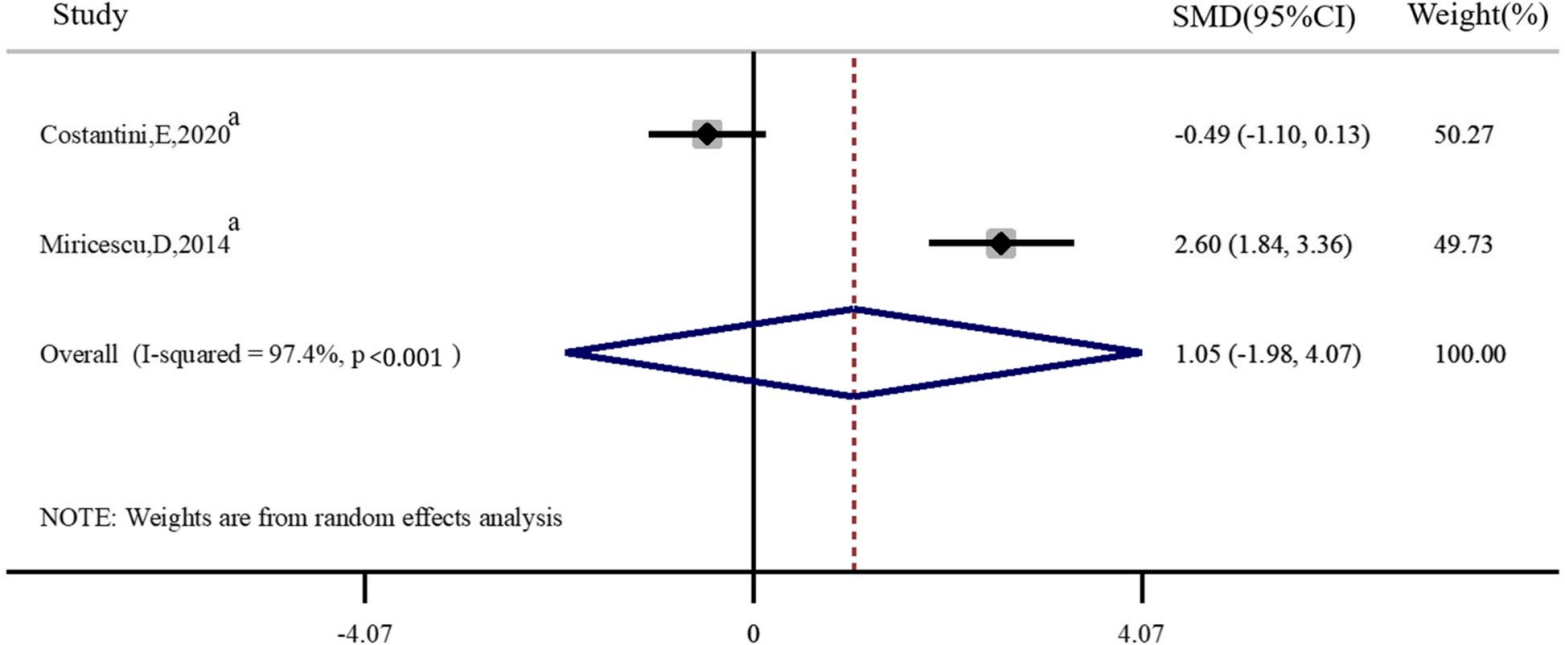
Forest plot of studies evaluating saliva 25(OH)D levels in individuals with and without periodontitis. SMD: standardized mean difference; a: case–control study

As shown in Fig. 5. Two articles [22, 38]compared the difference in saliva 25(OH)D levels between periodontitis and healthy individuals, and there was no significant difference in saliva 25(OH)D levels with periodontitis compared to normal population (*SMD* = 1.05, 95%*CI* 1.98 ~ 4.07, *P* = 0.50).

Due to the large heterogeneity among the studies, sensitivity analysis was conducted by eliminating study one by one. And the results of sensitivity analysis showed that excluding any study did not change the direction of the outcome, which indicated that the study was relatively stable (Supplementary Material Figure S1).

### Changes in vitamin D levels after SRP + vitamin D/SRP treatment of periodontitis

**Fig. 6:**
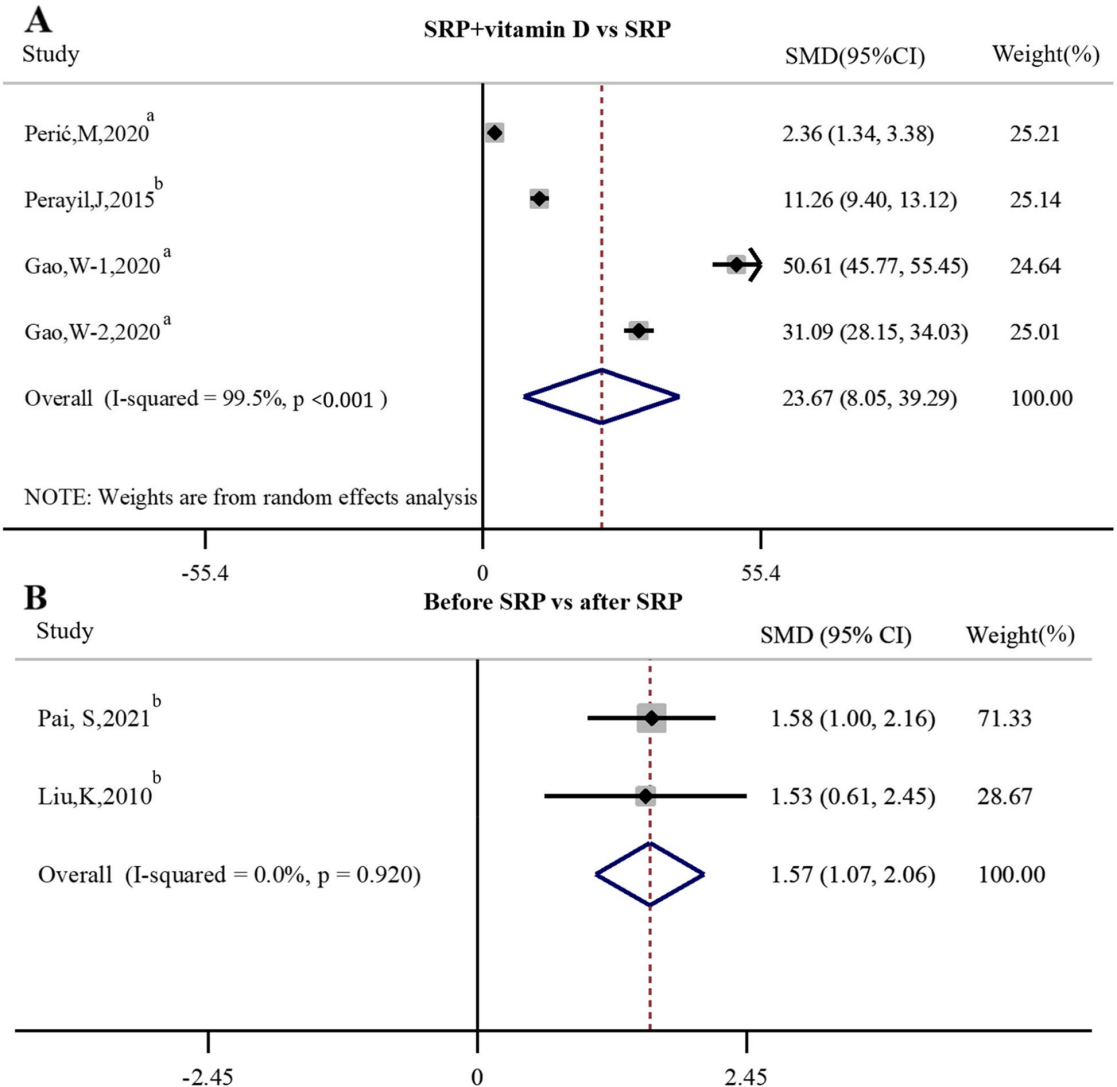
Forest plot of studies evaluating the effect of SRP + vitamin D versus SRP (before SRP versus after SRP). **A** SRP + vitamin D VS SRP. **B** Before SRP VS after SRP. Gao, W-1: Gao, W-2000 IU/d vitamin D; Gao, W-2: Gao, W-1000 IU/d vitamin D; SRP: scaling and root planing. SMD: standardized mean difference; a: RCT; b: Non-RCT

There were three intervention trails [33, 40, 41] assessed the effects of SRP + vitamin D versus SRP on serum vitamin D levels in individuals with periodontitis, and the result of Meta-analysis showed that SRP + vitamin D was beneficial to increase the serum vitamin D concentration in individuals with periodontitis compared to SRP (*SMD* = 23.67, 95%*CI* 8.05 ~ 32.29, *P* = 0.003). At the same time, two intervention trails [36, 39] studied the effects of SRP on serum vitamin D levels in individuals with periodontitis, and the result showed that there was a statistically significant difference in serum vitamin D levels of individuals with periodontitis between before and after SRP (*SMD* = 1.57, 95%*CI* 1.07 ~ 2.06, *P* < 0.01). As shown in Fig. 6.

### Meta-analysis of SRP + vitamin D on periodontal clinical parameters

**Fig. 7:**
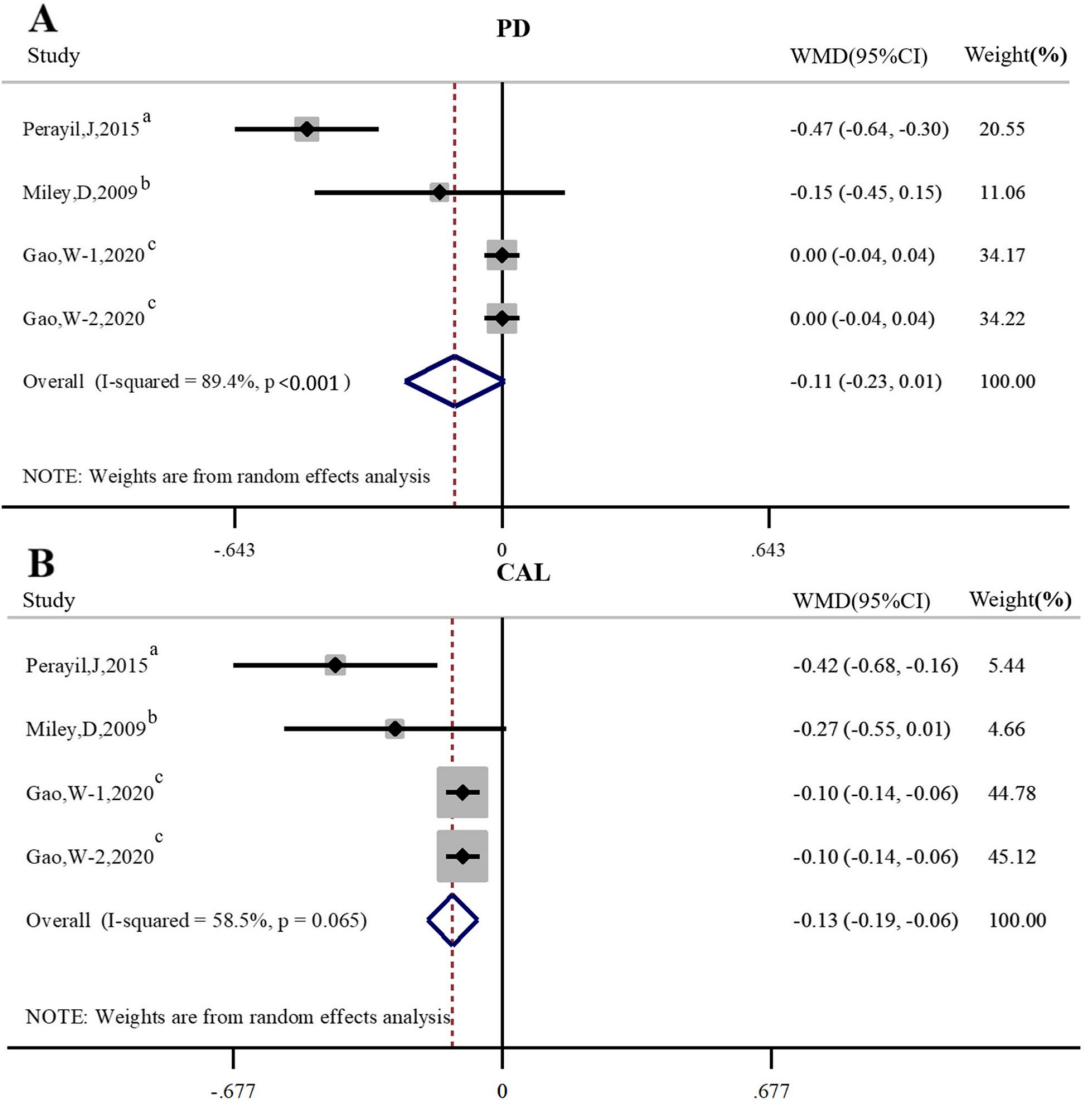
Forest plot of studies evaluating the effect of SRP + vitamin D on PD and CAL compared to SRP alone. **A** Probing depth (PD). **B** Clinical attachment level (CAL). Gao, W-1: Gao, W-2000 IU/d vitamin D; Gao, W-2: Gao, W-1000 IU/d vitamin D; WMD: weighted mean difference;a: Non-RCT; b: cross-sectional study; c: RCT

There were three studies [33, 37, 40] evaluated the effects of SRP + vitamin D on PD compared to SRP alone, and no significant difference was found in PD between SRP + vitamin D and SRP in individuals with periodontitis (*WMD* = -0.11, 95%*CI* -0.23 ~ 0.01,* P* = 0.07). There were three studies [33, 37, 40] evaluated the effects of SRP + vitamin D on CAL compared to SRP alone. The results of Meta-analysis showed that vitamin D as an adjunct to SRP was demonstrated to be beneficial to reduce CAL in individuals with periodontitis (*WMD* = -0.13, 95%*CI* -0.19 ~ -0.06, *P* < 0.01). As shown in Fig. 7.

There were two studies [37, 40] evaluated the effects of SRP + vitamin D on GI compared to SRP alone, and no significant difference was found in GI between SRP + vitamin D and SRP in individuals with periodontitis (*WMD* = -0.55, 95%*CI* -1.11 ~ 0.01, *P* = 0.05). There was one study [33] evaluated the effects of SRP + vitamin D (2000 IU/d, 1000 IU/d) on BI compared to SRP alone. The results of Meta-analysis showed that there was no significant difference in GI between SRP + vitamin D and SRP in individuals with periodontitis (*WMD* = -0.05, 95%*CI* -0.15 ~ 0.05, *P* = 0.32) (Supplementary Material Figure S2).

## Publication bias

Publication bias was tested by Begg's test and Egger's test by Stata 14.0 software. All *P*-values from the Begg’s Test and Egger’s test were greater than 0.05, the results did not show any evidence of publication bias. So it was believed that there was no publication bias among the studies of SRP + vitamin D on periodontal clinical parameters (Supplementary Material Table S4). Also, the funnel plot did not reveal any significant publication bias regarding serum vitamin D levels in individuals with and without periodontitis (Supplementary Material Figure S3).

## Discussion

The Meta-analysis included both intervention and observational studies of varying quality, which will influence the interpretation of results. And high quality scores for both cross-sectional studies [37, 42] and case–control studies [17, 22, 30–32, 34, 35, 38, 43]. Two RCTs [33, 41] were at low risk of bias in all aspects except other bias, and three Non-RCTs [36, 39, 41] had a low risk of bias in terms of selection of participants, intervention classification, outcome measurement and selection of reported result, however, one of the studies [36]had a moderate risk of bias in confounding factors and deviation from intended intervention, two studies [39, 41] had a serious risk of bias in missing data. Therefore, more high-quality literature is required to validate this result in the future.

The results of this meta-analysis showed that SRP + vitamin D could significantly reduce CAL compared to SRP alone. And similar to Machado V, et al. [16], this systematic review supported that periodontitis was associated with lower serum vitamin D levels compared to normal population, while there was no significant difference in serum or saliva 25(OH)D levels between periodontitis individuals and normal population. Although Machado V, et al. [16] performed descriptive analysis in vitamin D supplementation during nonsurgical periodontal therapy, there was a lack of quantitative evidence to support the effect of vitamin D in adjunctive periodontal nonsurgical treatments such as SRP. Therefore, this Meta-analysis compared the effect of SRP + vitamin D and SRP alone in the treatment of periodontitis, and it was found that SRP + vitamin D could significantly reduce CAL compared to SRP alone, but had no meaningful effect on PD, GI and BI.

At the same time, considering the large heterogeneity among the results of the Meta-analysis, stata14.0 software was used for sensitivity analysis through one-by-one elimination, which found that none of the results changed the direction, indicating that the included studies were stable. And subgroup analysis of study types, publication year, samples source (serum or saliva), measurement method of vitamin D (Elisa, HPLC–MS, CLIA), country, and gender showed that none of the above factors were sources of heterogeneity for the included studies. Given the lack of evidence of clinical heterogeneity, this Meta-analysis was performed using random-effects models.

This Meta-analysis found there were statistically significant differences in serum vitamin D levels between individuals with periodontitis and healthy population, that is to say, periodontitis was associated with lower serum vitamin D levels compared with normal population. 25(OH)D is regarded as the best indicator of vitamin D status [44]. However, the present study found that there were no differences in serum or saliva 25(OH)D levels between periodontitis individuals and normal periodontal individuals. And this result was supported by the Millen et al., Antonoglou et al., and Amaliya et al. [45–47]. On the contrary, other studies have shown that plasma 25(OH)D levels were generally higher in individuals with periodontitis than in healthy population [38, 48], which was not consistent with the result of this study. This might be due to the fact that stages and grades of periodontitis in previous studies was different from that of the present study. In addition, the sample source of this study was different from that of previous studies, that is to say, the findings of this study were mainly based on serum samples, whereas the results of previous studies were primarily based on plasma or saliva samples, which might be partially responsible for the difference in results.

The results of this Meta-analysis showed that either SRP alone or SRP combined vitamin D could increase the serum vitamin D concentrations in individuals with periodontitis. In addition, SRP + vitamin D was helpful to improve periodontal clinical indicators. And the Meta-analysis demonstrated that SRP + vitamin D reduced CAL compared to SRP, although there was no significant difference in the improvement of PD, GI, and BI between SRP + vitamin D and SRP. Studies have shown that periodontitis is an inflammatory disease closely related to autoimmune regulation, and early recognition of early biomarkers of periodontitis, such as Transforming Growth Factor-β1 (TGF-β1) and Vascular endothelial growth factor (VEGF), is crucial for the treatment and prognosis of periodontitis [49, 50]. Vitamin D has immunomodulatory, anti-inflammatory and anti-proliferative effects, and plays an important role in bone metabolism, alveolar bone resorption and prevention of tooth loss [51–53]. Vitamin D can reduce gingival inflammation and promote wound healing after periodontal surgery by strengthening the antibacterial defense of gingival epithelial cells, which is an important supplement for the prevention of periodontal disease [51]. Thus, SRP + vitamin D could effectively reduce CAL in individuals with periodontitis compared to SRP, this result was in accordance with Perayil et al., Hiremath et al., and Mishra et al. [40, 54, 55], these studies found that vitamin D supplements had a dose-dependent anti-inflammatory effect in periodontitis or gingivitis individuals. However, there was no significant difference in the improvement of PD, GI, and BI between SRP + vitamin D and SRP, this might be attributed to the fact that SRP can reduce inflammatory burden by removing subgingival calculus and biofilm deposits in order to create a biocompatible root surface [56],and inadequate dose and duration of vitamin D supplementation may not have contributed to the statistical difference between SRP + vitamin D and SRP.

In a preliminary cohort study, similar to this systematical review, periodontal individuals with higher plasma vitamin D levels had lower PD and CAL [57]. Another study indicates vitamin D status in periodontal individuals was negatively correlated with GI [58]. In addition, vitamin D has a positive effect as a supplement to periodontal wound healing after non-surgical periodontal treatment, and an intervention trial showed that vitamin D supplementation contributed to improving CAL and PD after SRP [54, 59]. However, SRP + vitamin D only had a positive effect on improving CAL, but it had no advantage in PD,GI, and BI compared to SRP in the present study, this might be due to the small number and low quality of the included intervention studies. Therefore, higher quality studies are required to test the relationship between them in the future.

This study has the following shortcomings. Firstly, although a comprehensive search of five major English databases was conducted, all the included studies were in English, and there was a lack of published studies in other languages, as well as a lack of grey literature database search, which may cause publication bias to some extent. Secondly, there was a great deal of heterogeneity among studies, and the sources of clinical heterogeneity of some outcome indicators were still not fully explained by subgroup analysis and sensitivity analysis. Thirdly, due to the limited number of high-quality studies (RCTs) evaluating the relationship between vitamin D and periodontitis, observational studies and intervention studies meeting the inclusion and exclusion criteria were included in this meta-analysis in order to more comprehensively analyze the relationship between vitamin D and periodontitis. Although confounding factors were strictly controlled for observational studies and subgroup analysis was performed based on study type. However, the heterogeneity results could not be changed, and these bias factors would inevitably affect the results of the meta-analysis. Therefore, the interpretation of the results of this study should be cautious.

## Conclusion

In summary, vitamin D plays a positive role in the adjuvant treatment of individuals with periodontitis. Therefore, vitamin D in combination with nonsurgical periodontal therapy such as SRP has a practical application of value on the prevention and treatment of periodontitis in clinical practice. However, Due to the small number of included studies, high heterogeneity, and lack of clear explanation of the source of heterogeneity, the actual effect of vitamin D combined with SRP in periodontitis in this study needs to be carefully interpreted, and a larger sample size and high- quality studies are still required to confirm it in the future.

## Availability of data of data and materials

All data generated or analysed during this study are included in this published article and its supplementary information files. Further inquiries can be directed to the corresponding author.

## Supplementary Information

**Additional file 1:**
