# Supplementary material for: Association of vitamin D in individuals with periodontitis: an updated systematic review and meta-analysis
Source: BMC Oral Health. 2023 Jun 13;23:387. doi: 10.1186/s12903-023-03120-w (PMC10265775; doi:10.1186/s12903-023-03120-w)
Supplement: Supplementary file 1 — Additional file 1: [file 12903_2023_3120_MOESM1_ESM.pdf]

Association of vitamin D in individuals with periodontitis: An Updated Systematic Review and Meta-analysis

Fangfang Liang [1, 2], Yuanzhu Zhou [1], Zhenyu Zhang [3], Zheng Zhang [1, 2], and Jing Shen [1, 2]

[1] *Tianjin Stomatological Hospital, School of Medicine, Nankai University, Tianjin, 300000, China*

[2] *Tianjin Key Laboratory of Oral and Maxillofacial Function Reconstruction, Tianjin 300041, China*

[3] *The School of Pharmacy, Jiamusi University, Jiamusi 154007, China*

Fangfang Liang, Yuanzhu Zhou, and Zhenyu Zhang contributed equally to this work.

Corresponding Author:

Zheng Zhang, Email address: zhangzheng@nankai.edu.cn,

Jing Shen, Email address: shenjing611@163.com

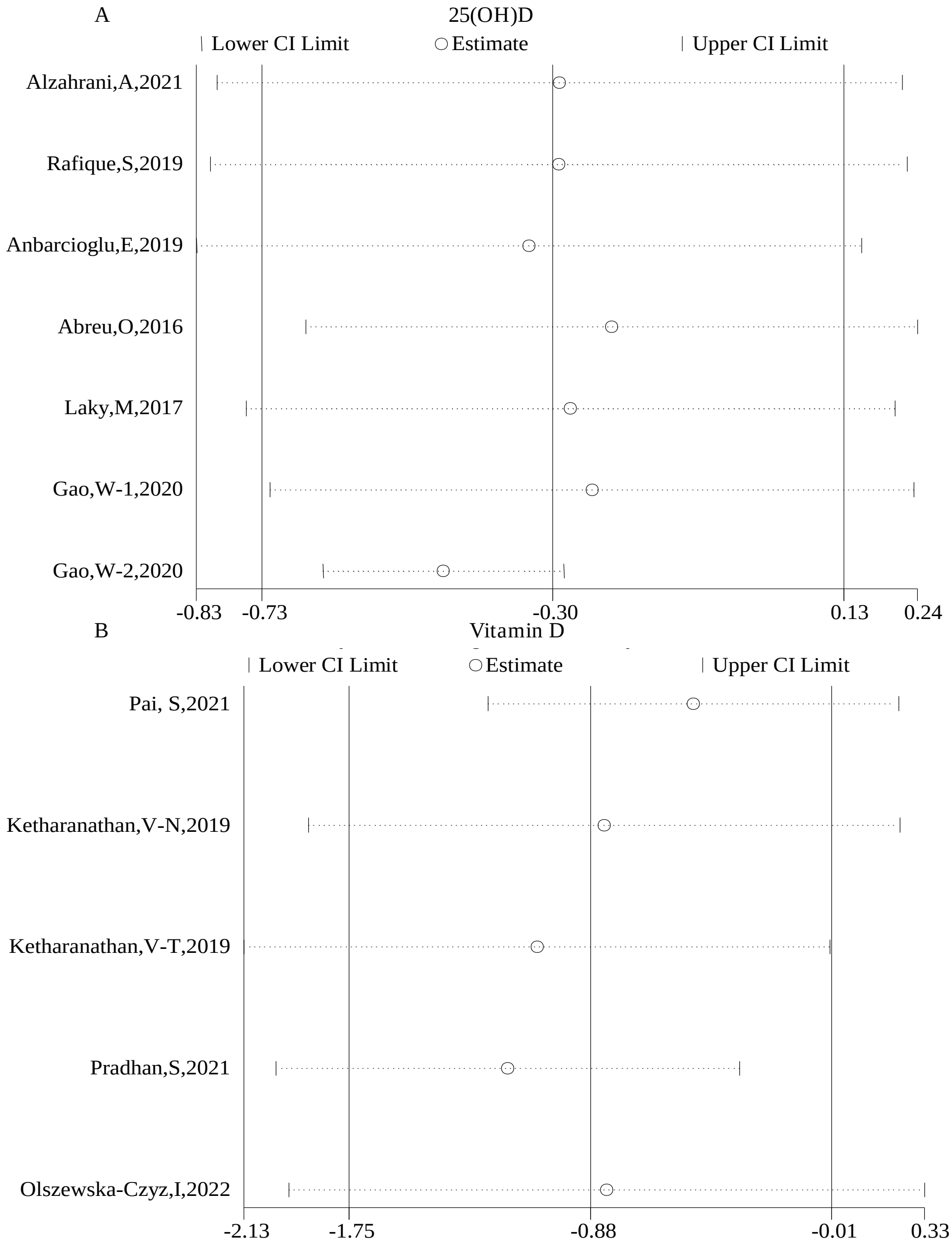

Figure S1. Sensitivity analysis of relationship between serum 25(OH)D/vitamin D and periodontitis.

(A)Serum 25(OH)D levels. (B)Serum vitamin D levels. Gao, W-1: Gao, W-2000 IU/d vitamin D; Gao, W-2: Gao,W-1000IU/d vitamin D; Ketharanathan,V-N: Ketharanathan,V-Norwegian;Ketharanathan,V-T: Ketharanathan, V-Tamil.

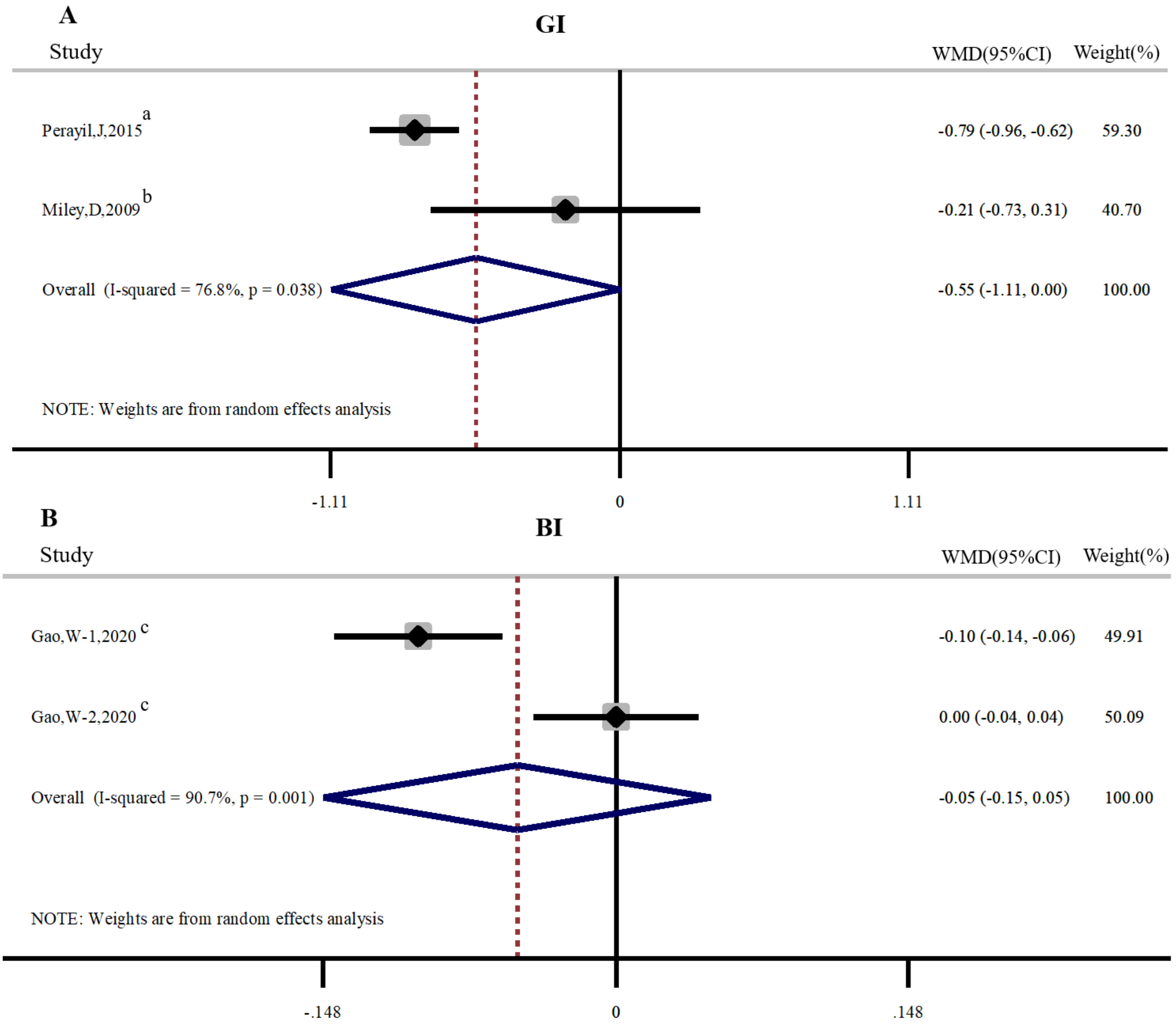

Figure S2. Forest plot of studies evaluating the effect of SRP+vitamin D on GI and BI compared to SRP alone. (A)Gingival index (GI). (B)Bleeding index (BI). Gao, W-1: Gao, W-2000 IU/d vitamin D; Gao, W-2: Gao,W- 1000IU/d vitamin D; WMD: weighted mean difference; a: Non-RCT; b: cross-sectional study; c: RCT.

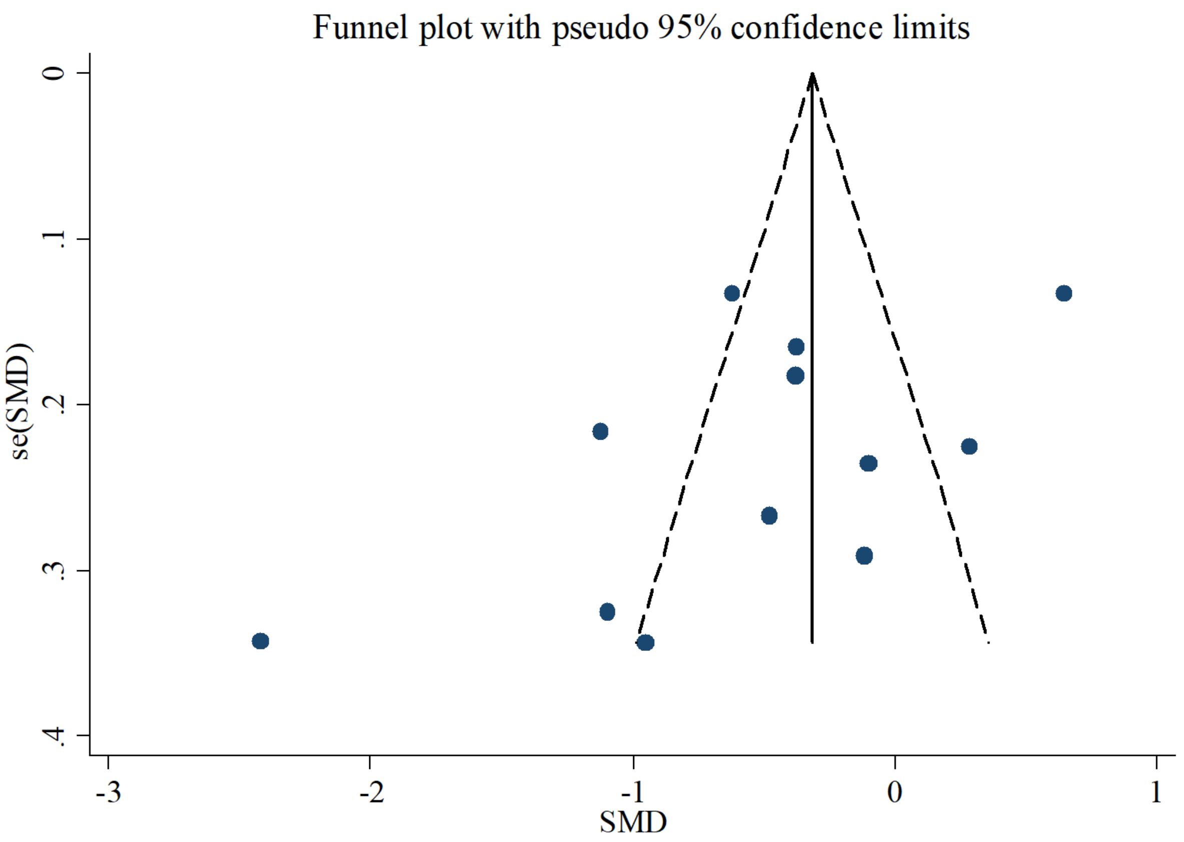

Figure S3. Funnel plot of studies evaluating serum 25(OH)D/vitamin D levels in individuals with and without periodontitis. SMD:standardized mean difference.

Table S1. The risk of bias in non-randomized studies of intervention (ROBINS-I) tool for assessing the quality of non-RCTs

Study	confounding	selection of participants	intervention classification	deviation from intended intervention	missing data	outcome measurement	selection of reported result
Liu,K(36),2010	Moderate	Low	Low	Moderate	Serious	Low	Low
Pai, S(39),2021	NI	Low	Low	Moderate	Serious	Low	Low
Perayil,J(40),2015	NI	Low	Low	Low	Low	Low	Low

NI:No Information.

Table S2. The Newcastle–Ottawa Scale (NOS) for assessing the quality of case-control studies

Study	Case definition	Representa tiveness	Selection of controls	Definition of controls	Comparabi lity	Ascertainm ent of	Same method	non-response	score
Olszewska-Czyz,I(17),2022	1	1	1	1	2	0	1	1	8
Costantini,E(22),2020	1	1	1	1	1	1	1	1	8
Abreu,O(30),2016	1	1	1	1	2	1	1	1	9
Alzahrani,A(31),2021	1	1	1	1	2	0	0	1	7
Anbarcioglu,E(32),2019	1	1	1	1	2	1	1	1	9
Ketharanathan,V(34),2019	1	1	1	1	2	0	1	1	8
Laky,M(35),2017	1	1	1	1	2	1	1	1	9
Miricescu,D(38),2014	1	1	1	1	1	1	1	1	8
Rafique,S(43),2019	1	1	1	1	2	1	1	1	9

Table S3. Agency for Healthcare Research and Quality (AHRQ) for assessing the quality of cross-sectional studies

Study	1	2	3	4	5	6	7	8	9	10	11	score
Miley,D(37),2009	1	1	0	1	0	1	1	1	1	1	0	8
Pradhan(42),S·2021	1	1	1	1	0	0	1	1	1	1	1	9

Note:Specific items are available from: https://www.ncbi.nlm.nih.gov/books/NBK35156/

Table S4. Publication bias on periodontal clinical parameters

Outcome	Begg's Test		Egger's Test	
	Z	*P*	*t*	*P*
PD	1.02	0.308	-1.75	0.223
CAL	1.02	0.308	-3.95	0.059
GI	0.00	1.000	-	-
BI	0.00	1.000	-	-

PD:Probing depth; CAL:Clinical attachment level; GI:Gingival index; BI:Bleeding index.
